# Supplementary material for: DMRforPairs: identifying Differentially Methylated Regions between unique samples using array based methylation profiles
Source: BMC Bioinformatics. 2014 May 15;15:141. doi: 10.1186/1471-2105-15-141 (PMC4046028; doi:10.1186/1471-2105-15-141)
Supplement: Additional file 2 — DMRforPairs output for the comparison of A431-MCF7 and NA17018-NA17105. Please start from the HTML files in each folder. Available via the BMC Bioinformatics website. [file 1471-2105-15-141-S2.zip › 1394847754114233_MOESM2_ESM/A431_MCF7/figures/37317.pdf]

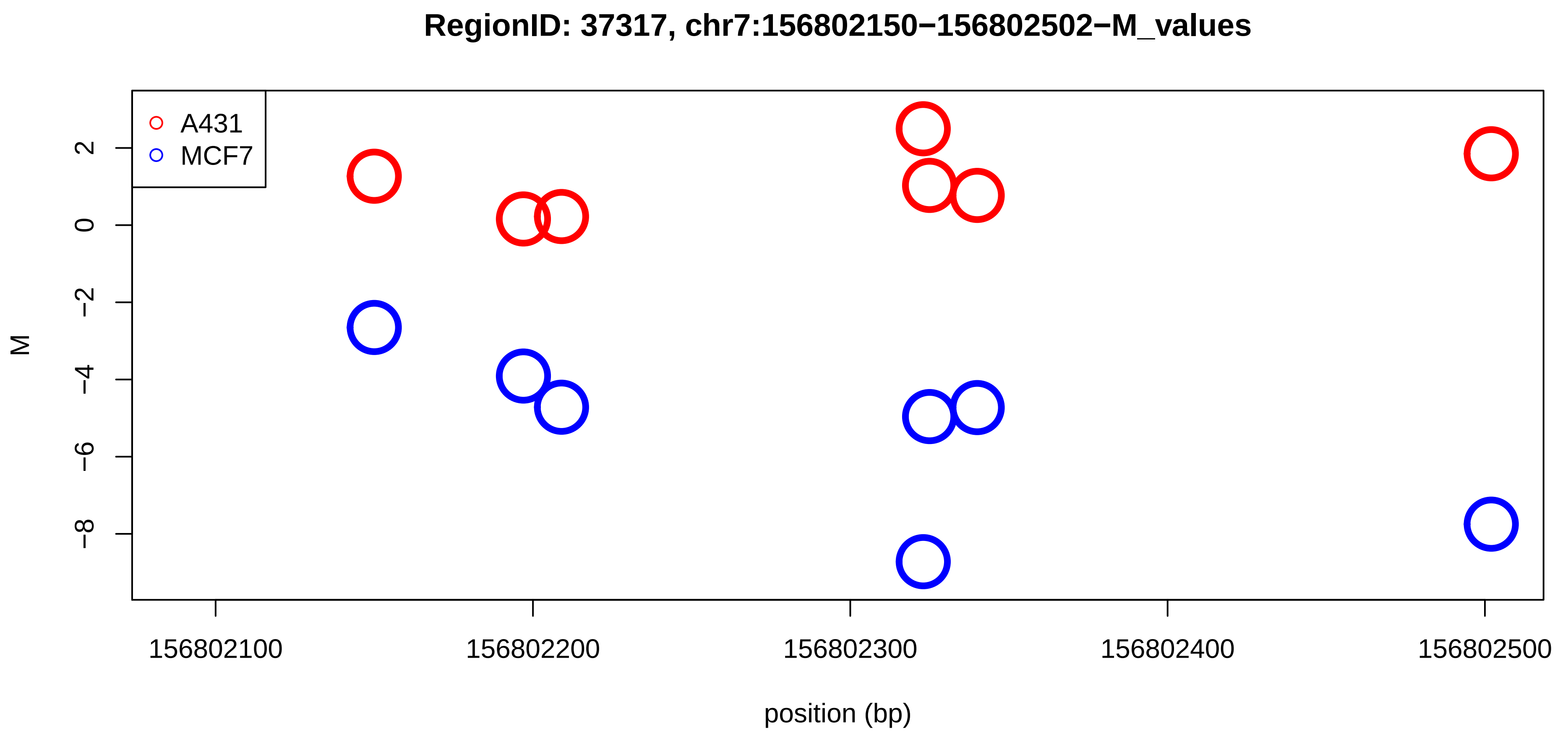
RegionID: 37317, chr7:156802150−156802502−M_values
A431
MCF7
M
position (bp)
156802100
156802200
156802300
156802400
156802500

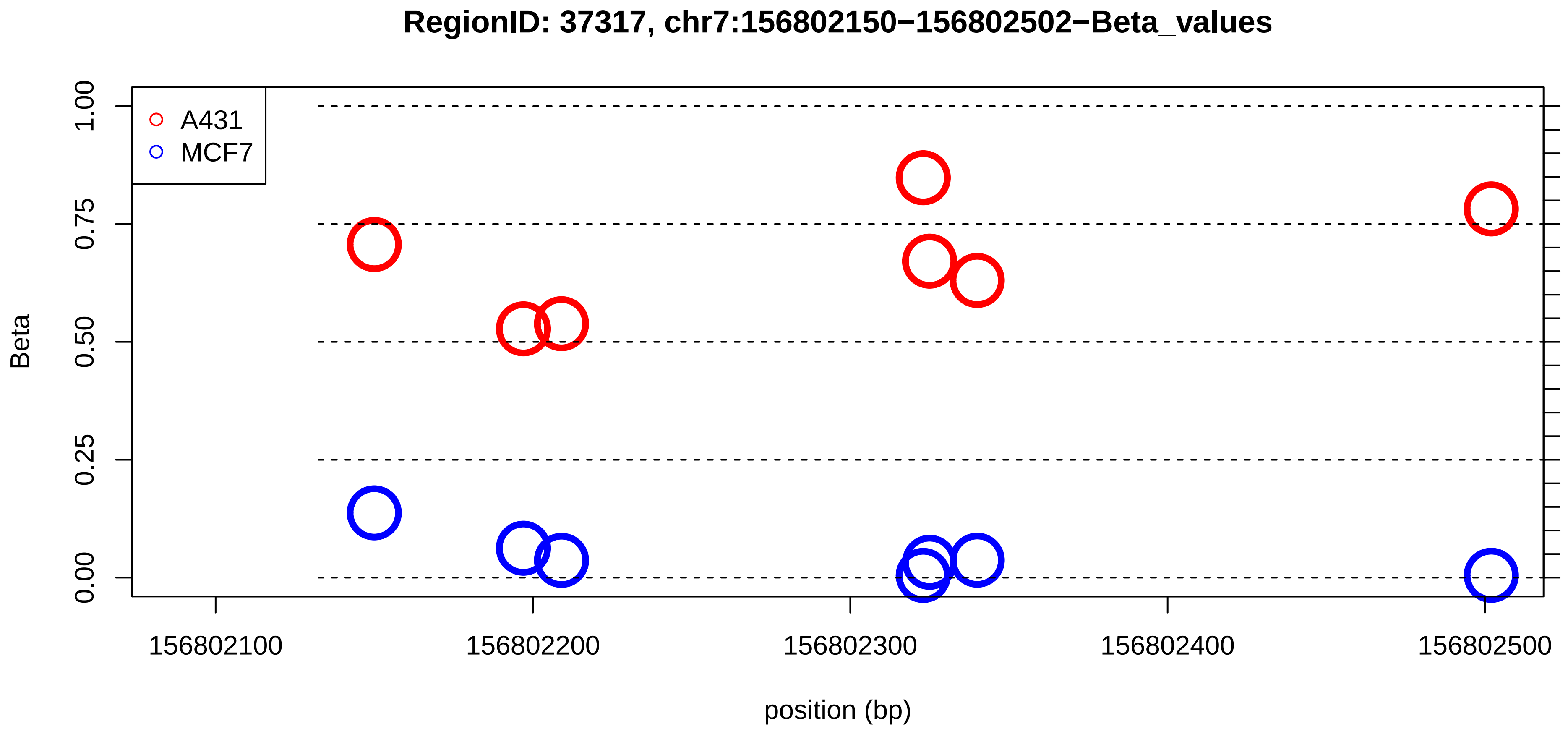
RegionID: 37317, chr7:156802150−156802502−Beta_values
A431
MCF7
Beta
position (bp)
156802100
156802200
156802300
156802400
156802500

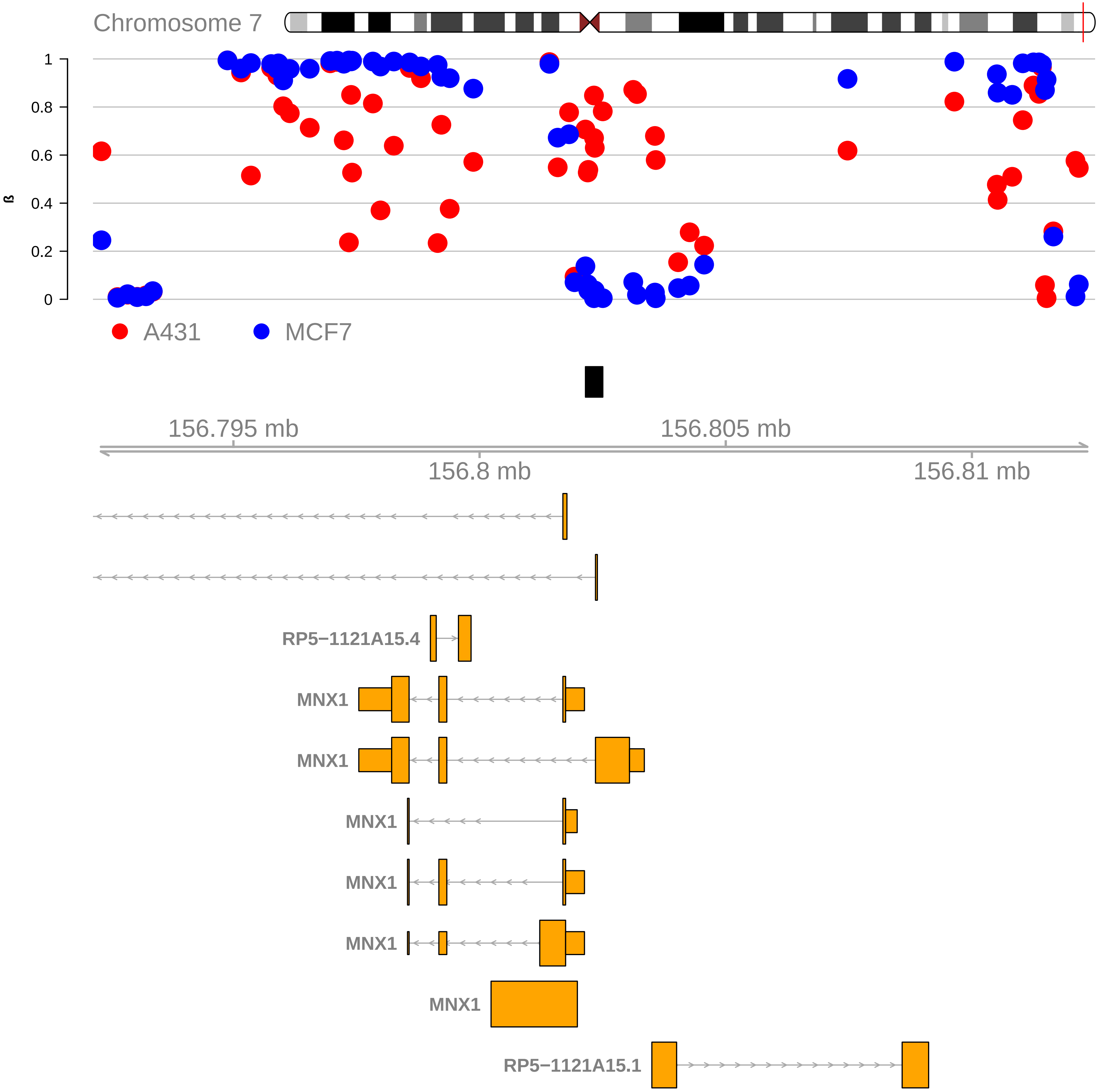
Chromosome 7
1
0.8
0.6
0.4
0.2
0
ß
A431
MCF7
156.795 mb
156.805 mb
156.8 mb
156.81 mb
RP5−1121A15.4
MNX1
MNX1
MNX1
MNX1
MNX1
MNX1
RP5−1121A15.1
